# miR-218 Inhibits Mitochondrial Clearance by Targeting PRKN E3 Ubiquitin Ligase

**DOI:** 10.3390/ijms21010355

**Published:** 2020-01-05

**Authors:** Anthea Di Rita, Teresa Maiorino, Krenare Bruqi, Floriana Volpicelli, Gian Carlo Bellenchi, Flavie Strappazzon

**Affiliations:** 1 IRCCS Fondazione Santa Lucia, 00143 Rome, Italy; antheadirita@gmail.com (A.D.R.); maiorino.teresa@gmail.com (T.M.); krenare.bruqi@gmail.com (K.B.); bellenchi@igb.cnr.it (G.C.B.); 2 University of Rome Tor Vergata, 00133 Rome, Italy; 3 Institute of Genetics and Biophysics “Adriano Buzzati Traverso”, CNR, 80131 Naples, Italy; floriana.volpicelli@unina.it; 4 Department of Pharmacy, University of Naples Federico II, 80131 Naples, Italy; 5 Department of Systems Medicine, University of Rome Tor Vergata, 00133 Rome, Italy

**Keywords:** microRNA, mitochondria, miR-218, PARKIN/PRKN, mitophagy

## Abstract

The selective elimination of dysfunctional mitochondria through mitophagy is crucial for preserving mitochondrial quality and cellular homeostasis. The most described mitophagy pathway is regulated by a positive ubiquitylation feedback loop in which the PINK1 (PTEN induced kinase 1) kinase phosphorylates both ubiquitin and the E3 ubiquitin ligase PRKN (Parkin RBR E3 ubiquitin ligase), also known as PARKIN. This event recruits PRKN to the mitochondria, thus amplifying ubiquitylation signal. Here we report that miR-218 targets PRKN and negatively regulates PINK1/PRKN-mediated mitophagy. Overexpression of miR-218 reduces PRKN mRNA levels, thus also reducing protein content and deregulating the E3 ubiquitin ligase action. In fact, following miR-218 overexpression, mitochondria result less ubiquitylated and the autophagy machinery fails to proceed with correct mitochondrial clearance. Since mitophagy defects are associated with various human diseases, these results qualify miR-218 as a promising therapeutic target for human diseases.

## 1. Introduction

Autophagy is an important eukaryotic process involved in the lysosomal degradation of cytosolic components in both physiological and pathological conditions. During autophagy, some vesicles named autophagosomes engulf a number of different cargoes and then fuse with lysosomes for subsequent recycling of their content [1]. Selective elimination of undesired or dysfunctional mitochondria through autophagy is called mitophagy [2]. This evolutionary-conserved mechanism removes damaged mitochondria in order to reduce reactive oxygen intermediates that are known to participate in inflammation, genotoxic stress, promotion of tumorigenesis, and aging [3]. Accordingly, defects of the mitophagic pathway contribute to neurodegenerative or inflammatory diseases and cancers. However, mitophagy inhibition may also be beneficial in some human diseases. For instance, it has been demonstrated that melatonin exerts neuroprotective effects against glutamate-induced excitotoxicity by reducing mitophagy-related oxidative stress and maintaining mitochondrial function [4]. In the context of cancer, mitophagy was initially thought to be an onco-suppressor that helps in maintaining cellular homeostasis [5], but now emerging evidence indicates that mitophagy inhibition may favor cancer cell survival by eliminating damaged mitochondria and reducing, in this way, mitochondrial reactive oxygen species [6]. The mitophagy manipulation is thus crucial for cancer therapy. During mitophagy, two post-translational modifications, such as ubiquitylation and phosphorylation, orchestrate an efficient mechanism for mitochondria specific elimination [7]. To date, the most characterized mitophagy pathway is regulated by the PINK1 (PTEN induced kinase 1) protein. PINK1 is a serine-threonine kinase that phosphorylates both ubiquitin and the E3 ubiquitin ligase PRKN (Parkin RBR E3 ubiquitin ligase), also known as PARKIN, at serine 65 (S65). PRKN phosphorylation is necessary for recruiting and activating the E3 ubiquitin ligase activity on dysfunctional mitochondria [8,9,10]. This event leads PRKN to conjugate ubiquitin chains on outer mitochondrial membrane proteins. Ubiquitins are thus phosphorylated on S65 to further stimulate in a positive feedback loop PRKN recruitment and activation [11,12,13,14,15,16]. This rapid amplification of pS65-Ub chains on the mitochondria is fundamental to autophagy machinery recognition. In fact, some proteins, known as mitophagy receptors, bind ubiquitin chains and act as a bridge between the autophagosome marker MAP1LC3A/LC3 (microtubule associated protein 1 light chain 3 alpha) and the dysfunctional mitochondria. In PINK1/PRKN system, OPTN (Optineurin) and CALCOCO2/NDP52 (calcium binding and coiled-coil domain 2) are the primary mitophagy receptors [17,18,19,20]. Besides the PINK1/PRKN-dependent mitophagy, mammalian mitochondria selective clearance is also controlled by other proteins, which function in an alternative pathway, including BNIP3L (BCL2 interacting protein 3 like) [21,22], FUNDC1 (FUN14 domain containing 1) [23], AMBRA1 (Autophagy and Beclin 1 regulator 1) [24], BCL2L13 (BCL2 like 13) [25] or that can cooperate with the PINK1/PRKN system, such as AMBRA1 [26] and PHB2 (Prohibitin 2) [27].

In the last few years, studies have demonstrated the involvement of microRNAs (abbreviated miRNAs), which are small non-coding RNA molecules (containing about 22 nucleotidies), in a large spectrum of processes, including autophagy [28,29]. Moreover, it has been shown that miRNAs control also mitochondria selective autophagy. In fact, Barde and colleagues demonstrated that a persistent expression of miRNAs causes a hematopoietic-restricted deletion of Kap1 that inhibits mitophagy and retains mitochondria into erythroblasts [30]. In addition, a hypoxia-responsive miRNA, miR-137 targets both FUNDC1 and BNIP3L thus inhibiting hypoxia-induced mitophagy. Furthermore, miR-124 protects against spinal cord ischemia–reperfusion injury by probably stimulating mitophagy [31]. Concerning the PINK1-mediated mitophagy, two specific regulations by miRNA have been proposed: (1) in 2016, it was shown that miR-181 controls mitochondrial clearance by targeting the E3 ligase PRKN [32]; (2) in the same year, it was established that miR-27a and miR-27b target PINK1 and abrogate the mitophagy pathway [33].

As it has been reported that PRKN E3 ubiquitin ligase could be a putative target of miR-218 [34] and since regulations are emerging between microRNA action and mitochondria selective removal, we decided here to investigate a potential role of miR-218 in mitophagy mediated by PINK1 and PRKN.

We demonstrated that PRKN is a target of miR218 in human cells. Moreover, we discovered that following mitophagy induction, miR-218 expression is sufficient to reduce the level of PRKN protein, limiting its translocation to mitochondria and thus reducing its ability to potentiate the ubiquitylation signal requested for the PINK1/PRKN-dependent mitophagy. Finally, we demonstrated that miR-218 expression is sufficient to delay mitochondrial clearance following mitophagy induction.

These results define a novel role of miR-218 in mitophagy and highlight a novel negative mechanism for mitochondria selective removal regulation. miR-218 could thus be a novel molecular mechanism that could be hijacked to treat several diseases such as cancer and neurodegenerative pathologies.

## 2. Results

### 2.1. miR-218 Targets PRKN E3 Ubiquitin Ligase

**Figure 1:**
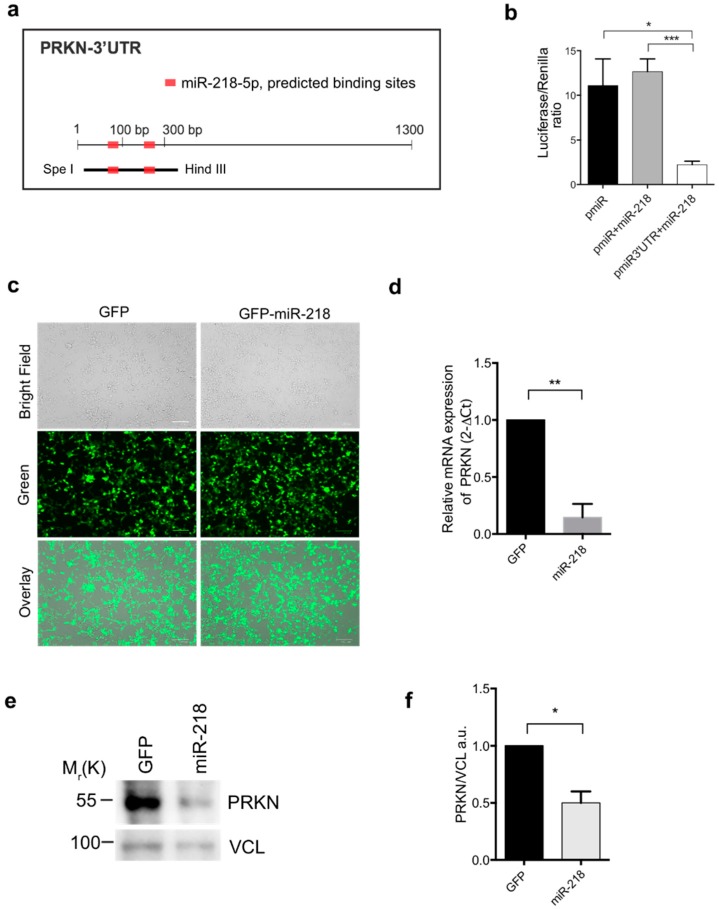
(**a**) Scheme of the 3’UTR (Untranslated region) of PRKN (Parkin RBR E3 ubiquitin ligase). The location of the two predicted binding sites for miR-218 are indicated in red. The region cloned in the pMiR Report miRNA expression vector is shown below the 3′UTR. The restriction enzymes used are indicated as well. (**b**) Luciferase assay. pMiR-Reports containing a portion of the PRKN 3’UTR (pMiR-3’UTR) or the empty pMiR vector (pMiR) were co-transfected in presence or absence of miR-218. All luciferase data have been normalized to the Renilla (RL-SV40) activity. The graph reports the Luciferase/renilla ratio. (**c**) Representative images of HEK293 cells transfected with GFP (Green Fluorescence Protein) or GFP-miR-218 vectors (left part) that were then analyzed by quantitative PCR (qPCR) in order to analyze the mRNA of PRKN (right part). Scale bar 100μm. (**d**) The graph shows the relative expression of PRKN, normalized on B2M as loading control. (**e**) Protein lysates of GFP or GFP-miR-218 transfected HEK293 cells were subjected to western blot analysis against PRKN antibody. (**f**) The graph reports the PRKN/VCL ratio. VCL/Vinculin is used as loading control. All data are representative of experimental triplicate (± s.e.m.). Statistical analysis was performed using Student t-test with Welch’s correction. * *p* < 0.05; ** *p* < 0.01; *** *p* < 0.001 M_r_(K) = relative molecular mass expressed in Kilo Dalton.

As it has been reported that PRKN may be a target of miR-218 [34], we first investigated whether miR-218 was able to target PRKN. In order to solve this question, by using target Scan software, we searched for putative miR-218 binding sites to the 3’ un-translated region (3’UTR) of PRKN. We did not find conserved matches in the human transcripts; however, two specific sites were identified in the 3’UTR of the mouse sequence for PRKN. These sites were located respectively at 80 bp and at 250 bp after the PRKN stop codon (Figure 1a).

To confirm the ability of miR-218 to bind this region we cloned a 350 bp portion of the PRKN 3’UTR, containing the two predicted binding sites, downstream the pMiR-REPORTER vector encoding the Renilla luciferase hereafter called pMiR-3’UTR (Figure 1b). Co-transfection of miR-218, with the pMiR-3’-UTR vector in HeLa cells results in a significant decrease in the luciferase activity, compared with empty vector (Figure 1b).

This result indicates that miR-218 is potentially able to down-regulate PRKN by binding specific sites at the 3’-UTR region.

The physiological role of such binding between miR-218 and PRKN has not been explored yet. In order to investigate, from a functional point of view, such an interaction, we overexpressed a vector encoding miR-218 comparing with an empty vector expressing the green fluorescent protein GFP alone in HEK293 cells, which are PRKN competent cells (Figure 1c). Then, we analyzed the mRNA levels of PRKN in the presence or absence of miR-218. As expected, we found that overexpression of miR-218 substantially reduced PRKN transcript levels (Figure 1d). Moreover, we analyzed the PRKN protein levels by performing a western blot analysis. As illustrated in Figure 1e, HEK293 cells transfected with a vector encoding miR-218 show a decrease in PRKN protein level compare to GFP-positive control cells (Figure 1e,f).

These results indicate that miR-218 down-regulates PRKN levels in HEK293 cells.

### 2.2. miR-218 Inhibits Mitochondrial Clearance

**Figure 2:**
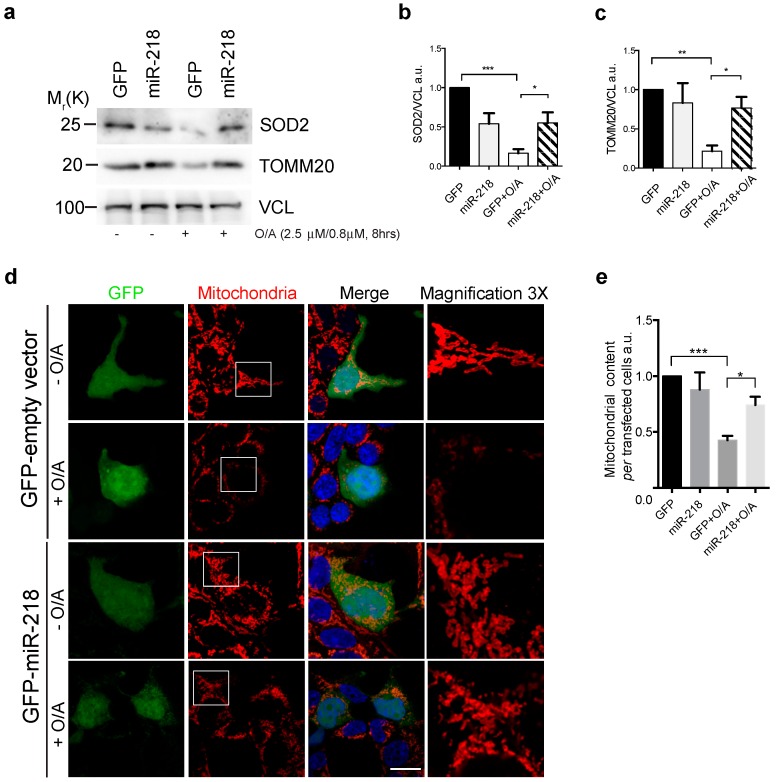
(**a**) HEK293 cells transfected with GFP or GFP-miR-218 vectors and then treated with O/A (Oligomycin and Antimycin A 2.5 μM, 0.8 μM, 8 h) were immunoblotted for the indicated antibodies. (**b**,**c**) The graphs show the SOD2 (superoxide dismutase 2) and TOMM20 (translocase of outer mitochondria membrane 20) protein level normalized on the VCL (Vinculin) loading control. (**d**,**e**) Representative immunofluorescence image and related graph in which GFP or GFP-miR-218 overexpressing HEK293 cells were immunostained with an anti-TOMM20 to detect mitochondria (red). Magnifications (3X) of the areas localized in the white frames are illustrated for each immunofluorescence. Scale bar, 10 μm. All data represent the mean of experimental triplicate (±s.e.m.). Statistical analysis was performed using One-Way ANOVA with Sidak’s correction. * *p* < 0.05; ** *p* < 0.01; *** *p* < 0.001. Mr(K) = relative molecular mass expressed in Kilo Dalton.

Since we demonstrated that PRKN is a novel target of miR-218 in HEK293 cells (Figure 1) and since PRKN is important for a correct amplification of the PINK1-mediated mitophagy, we hypothesized here that miR-218 expression was able to interfere with mitochondrial clearance by reducing PRKN levels. To test this hypothesis, HEK293 cells transfected with GFP or GFP-miR-218 vectors were treated with oligomycin and antimycin A (O/A), two agents commonly used to induce mitochondrial damage in order to activate mitophagy [13,20]. We next checked for mitophagy occurrence; to do this, we analyzed levels of two mitochondrial proteins, a common method used to monitor mitophagy. As expected, we observed a strong decrease of both SOD2/MnSOD (superoxide dismutase 2) and TOMM20 (translocase of outer mitochondria membrane 20) following mitophagy induction, suggesting that mitochondrial clearance occurs (Figure 2a–c). However, when we overexpressed miR-218, we found no evident reduction of mitochondrial proteins upon O/A treatment, suggesting that mitochondrial clearance was blocked by miR-218 overexpression. Then, we decided to strengthen our biochemical data by performing a confocal microscopy analysis. We confirmed that miR-218 was able to inhibit mitochondrial clearance following mitophagy stimulation. In fact, in GFP-transfected cells treated with O/A, we observed a huge decrease of mitochondrial content; however, when we over-expressed miR-218, no evident decrease was observed in transfected cells (Figure 2d,e).

Overall, these results suggest that miR-218 over-expression delays mitochondria selective removal following mitophagy induction.

### 2.3. miR-218 Inhibits Mitophagy by Reducing Prkn Expression and Function

**Figure 3:**
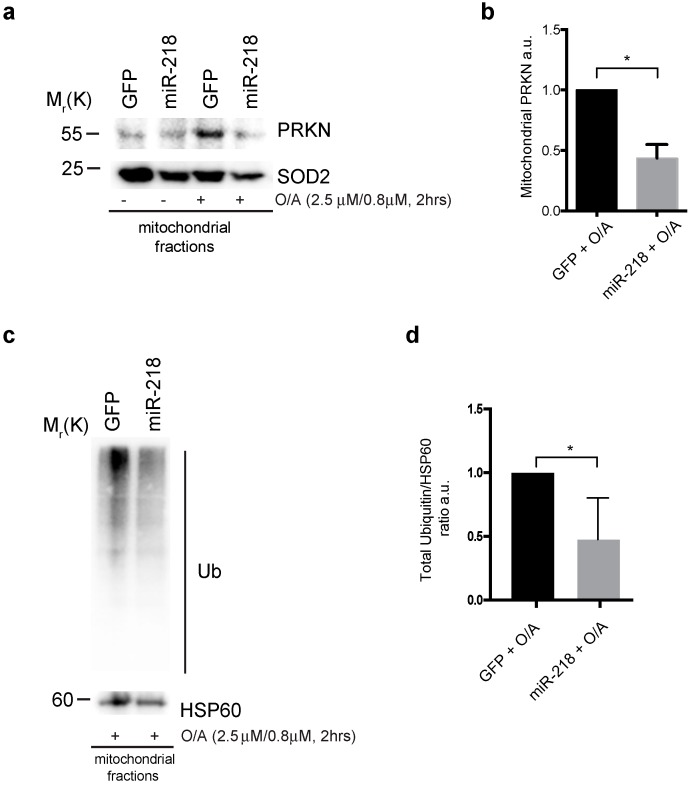
(**a**) HEK293 cells overexpressing GFP or GFP-miR-218 vectors were treated with O/A for 2 h and subjected to mitochondria purification. (**b**) The graph shows the mitochondrial PRKN normalized on the mitochondrial loading control. (**c**) Representative image of HEK293 cells transfected with GFP or GFP-miR-218 constructs and treated with O/A for 2 h in order to analyze total ubiquitin amount in mitochondria. (**d**) The graph shows total Ub (Ubiquitin) normalized on the mitochondrial protein HSP60 (Heat Shock Protein 60). All data represent the mean of three experiments (±s.e.m.). * *p* < 0.05. Statistical analysis was performed using Student t-test with Welch’s correction. Mr(K) = relative molecular mass expressed in Kilo Dalton.

Since we found that one of the main players of mitophagy pathway, PRKN, is a target of miR-218, and since this miRNA negatively controls mitochondria selective removal, we expected that miR-218 would delay mitochondrial clearance by targeting PRKN protein levels and thus affecting its known function in mitophagy. In order to address this point, we first evaluated the PRKN translocation to the mitochondria, a necessary event for a correct mitophagy induction. In fact, upon mitophagy stimulation, the cytosolic E3 ubiquitin ligase PRKN is known to be recruited to the mitochondria in order to amplify the ubiquitylation events necessary for the recruitment of mitophagy receptors [8,9,10]. To test this, HEK293 cells over-expressing GFP or GFP-miR-218 plasmids were treated with O/A in order to stimulate mitophagy. Cells were then subjected to mitochondria fractionation and immunoblotting analysis. As shown in Figure 3a,b we observed a reduction of mitochondrial PRKN comparing with the control condition following mitophagy induction. Since a reduction in PRKN mitochondrial level is evident, we hypothesized that, in the meantime, mitochondria could be less-ubiquitylated in that context. To verify this hypothesis, we transfected HEK293 cells with GFP or GFP-miR-218 constructs and we treated cells with O/A in order to induce mitophagy. We then performed a mitochondrial fractionation in which we analyzed ubiquitylation by measuring the amount of total ubiquitin in these extracts. The ubiquitylated mitochondrial proteins decreased upon miR-218 over-expression, suggesting that the PRKN-dependent ubiquitin amplification signal is reduced (Figure 3c,d).

Our findings reveal that miR-218 over-expression promotes a reduction of PRKN levels during mitophagy, leading to a decrease of PRKN translocation to mitochondria and most likely to a reduction of PRKN mitophagy activity.

### 2.4. miR-218 Inhibits Mitochondria Co-Localization with Autophagosomes

**Figure 4:**
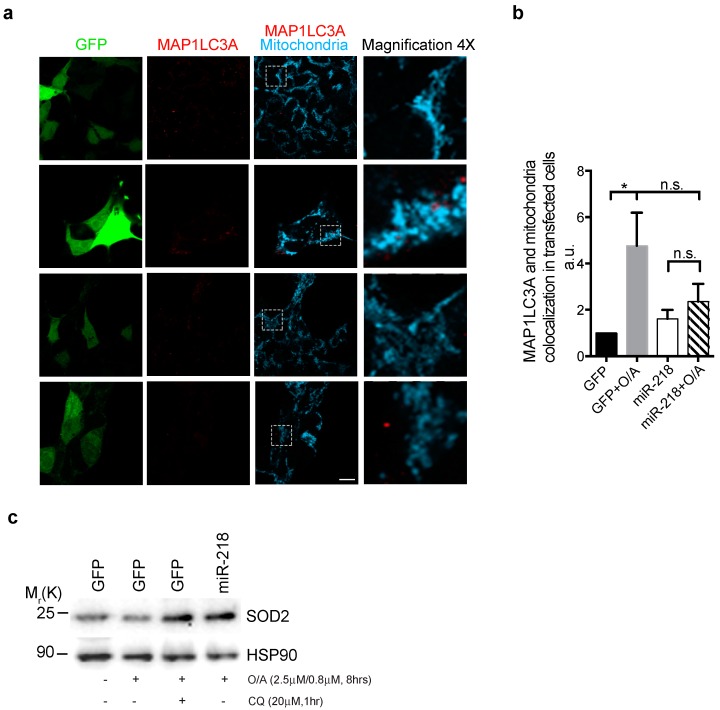
(**a**,**b**) Representative immunofluorescence image and related graph of HEK293 cells transfected with GFP or GFP-miR-218 vectors were treated with O/A for 5 h and immunostained with an anti-MAP1LC3A antibody (red) and an anti-TOMM20 (cyan) to detect mitochondria. Scale bar, 7 μm. The right panels report the magnifications (4X) of the images that are in the white dotted frames. Data represent the mean of three different samples (±s.e.m.) and are representative of experimental triplicate. * *p* < 0.05, n.s. (not significant). Statistical analysis was performed using One-Way ANOVA with Sidak’s correction. (**c**) Representative image of HEK293 cells transfected as in (**a**) and treated with O/A for 8 h in combination or not with the autophagosome-lysosome fusion inhibitor chloroquine (CQ), were immunoblotted for the indicated antibodies.

In order to demonstrate that miR-218 was able to negatively control a mitochondria clearance related to the autophagy pathway, we transfected HEK293 cells with vectors encoding GFP or GFP-miR-218 plasmids in order to determine the co-localization between the autophagy machinery substrate (mitochondria) and the autophagosome marker protein MAP1LC3A, upon mitophagy stimulation. As expected, we observed a high co-localization rate between mitochondria (in cyano) and MAP1LC3A-labelled autophagosomes (in red) in GFP transfected cells treated with O/A, by performing confocal microscopy analysis. Instead, by overexpressing miR-218, we observed no such increase in mitochondria and autophagosome co-localization (Figure 4a,b). Moreover, since during mitophagy, dysfunctional mitochondria, engulfed into autophagosomes, are delivered to lysosomes for degradation, we decided to verify whether miR-218 expression was efficient like the effect of Chloroquine (CQ), a well-known inhibitor of the fusion between autophagosomes and lysosomes [35]. To address this question, we transfected HEK293 cells with GFP or GFP-miR-218 and then we treated them or not with CQ, (Figure 4c). Upon mitophagy stimulation (O/A treatment), we detected a decrease of the mitochondrial marker SOD2 that was similarly rescued by overexpressing miR-218 or by blocking the fusion of autophagosomes and lysosomes with CQ.

Together these results demonstrate that miR-218 exerts an inhibitory effect on mitochondrial clearance through the autophagic pathway.

## 3. Discussion

An involvement of microRNAs in regulating highly controlled systems such as mitochondrial selective removal through autophagy pathway, also called mitophagy, has recently emerged [30,31,32,33]. Here, we analyzed the role of miR-218 in mitophagy, discovering its new target PRKN. Interestingly, we found that miR-218 is able to down-regulate PRKN mRNA in HEK293 cells demonstrating a novel function of miR-218. PRKN is fundamental in various neurodegenerative diseases, including Parkinson’s disease, Alzheimer’s disease, Amyotrophic Lateral Sclerosis and Huntington’s disease [36,37,38,39]. Furthermore, several studies have reported the ability of PRKN to protect neurons against neurotoxins and metallic ions [40]. Thus, maintaining an optimal PRKN expression and function appears to be crucial for neuron homeostasis.

In addition, PRKN is also involved in cancer regulation and the levels of its mRNA and protein are frequently down-regulated in ovarian and lung cancers [41,42,43,44,45,46].

Of note, the PRKN activity is strictly connected to mitochondria. In fact, in 2003, studies on *Drosophila* documented that, in absence of PRKN, mitochondria present abnormalities [47]. In addition, in 2008, Youle’s group demonstrated that PRKN regulates mitophagy in mammalian cells collaborating with PINK1 [48,49]. In this study, we showed that miR-218 action on PRKN could, at least, affect its function during the mitophagy process; in fact, miR-218 over-expression in HEK293 cells suppresses mitochondrial inhibitors-induced mitophagy by reducing the degradation of mitochondrial proteins. Besides the mitophagic process, mitochondrial-derived vesicles (MDVs) are known to be involved in the maintenance of mitochondrial homeostasis. MDVs are generated by selective incorporation of mitochondrial cargo into small vesicles, which transit to the lysosome for subsequent degradation. Interestingly, the MDVs transit to the lysosome is PINK1/PRKN-dependent [50]. Therefore, considering that both MDVs and mitophagy share common regulation pathways, our work places miR-218 as a novel attractive target in regulating the mitochondrial quality processes. Further studies are required to investigate a putative link between miR-218 and MDVs transit to lysosomes.

Preserving a correct mitophagy process is necessary for limiting reactive oxygen species (ROS) production, mitochondrial DNA mutations, metabolic inflexibility, and inflammation [51]. Upon PINK1 phophorylation, PRKN mediates the amplification of the ubiquitylation signal necessary for mitophagy receptors recruitment to mitochondria [8,9,10]. We identified miR-218 as a promising candidate to switch off PRKN gene, thus inhibiting mitophagy. Of note, autophagy inhibition has been widely accepted as a therapeutic strategy in cancer. In fact, autophagy regulators (e.g., chloroquine, hydrochloroquine) are already used in clinical studies on anticancer therapy. Now a general interest in mitophagy manipulation to improve cancer therapy is emerging. Indeed, inhibition of mitophagy (by targeting different stages of the autophagic/mitophagic process, genetically or pharmacologically) enhances drug sensitivity in several cases. For instance, the block of autophagosome formation and the inhibition of autophagosomes-lysosomes fusion were investigated [52,53,54]. The block of mitophagy by BNIP3L down-regulation enhanced doxorubicin sensitivity in colorectal cancer stem cells [55]. This said, few tools are available in order to specifically modulate the mitophagy pathway (and not only the general autophagy pathway). Here, we found that miR-218 is a novel powerful inhibitor of mitophagy through PRKN down-regulation. Interestingly, miR-218 was found significantly decreased in breast cancer tissues and negatively associated with Ki-67 [56]. The authors of this work demonstrated, in vitro, that over-expression of miR-218 induces apoptosis and decreases cell proliferation. In this regard, miR-218 mediated down-regulation of PRKN could be a potential mechanism that may contribute to apoptosis in breast cancer cells. Further studies are required to investigate this point.

Since nanoparticles offer incredible opportunities for cell specific controlled delivery of miRNAs in the treatment of cancer or neurodegenerative disorders [57], our findings thus provide a potential therapeutic strategy for cancer, such as breast cancer. Moreover, inhibition of miR-218 (e.g., AntagomiR) is expected to be beneficial in several neurodegenerative diseases in which the mitophagy process is not sufficiently efficient.

Interestingly, this E3 ubiquitin ligase is also the substrate of another miRNA, miR-181 [32]. It seems that both miR-218 and miR-181 have the same function in mammalian cells; as such, it would be interesting to understand if their functions are redundant or whether they could compensate each other. Since miR-218 is down-regulated in several cancers [58,59,60,61], we can suppose that expression of miR-181 could compensate these defects. However, when a strong stimulus promotes mitochondrial depolarization and thus powerful mitochondria selective removal is required, this pro-survival process can exceed in deregulated clearance that leads to apoptosis [62]. In this context, over-expressing miR-218 or generating a miR-218 agonist could turn out to be a promising weapon in order to combat mitophagy system’s dysregulation.

Finally, it has been recently demonstrated that despite its function in targeting PRKN during mitophagy, miR-181 control also genes implicated in mitochondrial biogenesis, functionality, and antioxidant response [63]. In analogy with this microRNA, it would be interesting to study whether miR-218 cover additional functions in controlling mitochondrial homeostasis beyond mitophagy.

In summary since PRKN and the mitophagy process play a pivotal role in neurodegeneration and tumor progression, our findings identify in miR-218 a potential therapeutic target for counteracting neurodegenerative and cancer diseases.

## 4. Materials and Methods

### 4.1. Cloning and Luciferase Assay

A portion of the 3′UTR sequence containing two predicted binding sites for miR-218 was cloned in the pMiR reporter vector by using the following oligonucleotides: forward: GCGCACTAGT-CGCACAACCTCAAGGGAAACTC (containing the cloning site for Spe I); reverse: GCGCAAGCTT-GCAGCGTTCCTCAGATCTCAAG (containing the cloning site for Hind III). The assay was performed by using the Luciferase Reporter Assay System (Promega, Milan, Italy), following the manufacturer’s instructions. The 3’UTR-containing pmiR-Report was co-transfected with the Tet-O-FUW miRNA-overexpressing vector and the rtTA-expressing vector in HeLa cells. A pRL-SV40 Renilla luciferase reporter vector (Promega, Milan, Italy) was also used to quantify the transfection efficiency. Firefly luciferase luminescent signal was normalized on the Renilla luciferase signal (Promega, Milan, Italy). Control experiments using the empty pMiR-Report vector in presence or absence of the microRNA were performed.

### 4.2. Cell Cultures and Transfection

Cells were cultured in Dulbecco’s modified Eagle’s medium (DMEM; GIBCO 41966-029, Monza, Italy) supplemented with 10% fetal bovine serum (FBS; Thermo Fisher Scientific, 10270-106, Monza, Italy) at 37 °C under 5% CO_2_. Transient transfections of expression plasmids into Hek293 cells were performed using TurboFect (Thermo Fisher Scientific, R0532, Monza, Italy).

### 4.3. Plasmids

The GFP-miR-218 construct was generated by cloning 400 bps encompassing the sequence for miR-218-1 into a Tet-O- FUW-Ires-GFP vector under the control of the tetracycline operator. Proper expression of mature miR-218 was tested by TaqMan (Thermo Fisher Scientific, Monza, Italy) assay as previously described [64]. Transfection was performed in combination with the rtTA transactivator supplied with doxycycline (2 mg/mL, Clontech, Mountain View, California, USA). An empty Tet-O-FW GFP vector was used as control.

### 4.4. Cell Treatment

HEK293 cells were treated with the combined treatment O/A (2.5 and 0.8 μM) for the indicated time. Autophagosome–lysosome fusion was blocked with CQ 20 μM for 1 h (Sigma-Aldrich, O9718, Milan, Italy).

### 4.5. Quantitative PCR (qPCR)

cDNA was isolated from Hek293 cells using the ReliaPrep RNA Cell Miniprep System (Promega, Milan, Italy) according to the manufacturer’s instruction. The quantitative analysis of PRKN was performed using quantitative real time polymerase chain reaction (PCR) for PRKN (Primer FW: GGGTCGTGAACAAACTGCCGATCATT; Primer RV: AGGAGCCCCGTCCTGGTTTT). We used B2M as housekeeping gene (Primer FW: CTCCGTGGCCTTAGCTGTG; Primer RV: TCTCTGCTGGATGACGTGAG). Each 20 μL reaction contained 5 μL of template, 1 μL of each primer (10 μM), 10 μL SensiFAST SYBR MIX 2× (Bioline, Milan, Italy) and 3 μL of nuclease-free water. The experiment was run in triplicate on a LC480 LightCycler (Roche, Milan, Italy). PRKN content was calculated with the ΔΔCt method after normalization on B2M.

### 4.6. Western Blot Analysis

Cells were rinsed in phosphate-buffered saline (PBS) on ice and lysed in RIPA (Radioimmunoprecipitation assay buffer) buffer supplemented with a protease inhibitor cocktail (Sigma-Aldrich, P8340, Milan, Italy), Na4VO3 0.1 mM (Sigma-Aldrich, S6508, Milan, Italy), NaF 1 mM (Sigma-Aldrich, S7920, Milan, Italy) and β-Glycerophosphate 5 mM (Sigma-Aldrich, G6376, Milan, Italy). Cell extracts were centrifuged at 15,000× *g* for 10 min at 4 °C. Protein concentrations were determined with the Bio-Rad Protein Assay Kit (Bio-Rad, 5000001, Milan, Italy). Cell extracts were separated by sodium dodecyl sulphate–polyacrylamide gel electrophoresis (SDS–PAGE). Primary antibodies used were: anti-SOD2 (Enzo-Lifesciences, Milan, Italy), anti-TOMM20 (Santa Cruz Biotechnology, Milan, Italy), anti-PRKN (Cell signalling, Milan, Italy), anti-VCL (Santa Cruz Biotechnology, Milan, Italy), anti-Ub (Santa Cruz Biotechnology, Milan, Italy), anti-Ub (KK2, Enzo-Lifesciences, Milan, Italy), anti-HSP60 (Santa Cruz Biotechnology, Milan, Italy). All uncropped images are illustrated in Supplementary Figure S1).

### 4.7. Immunofluorescence Analysis

Cells were washed in PBS and fixed with 4% paraformaldehyde in PBS for 10 min. After permeabilization with 0.4% Triton X-100 (Sigma-Aldrich, X-100, Milan, Italy) in PBS for 5 min, cells were incubated overnight at 4 °C with primary antibodies and 2% normal goat serum (Sigma-Aldrich, G9023). Primary antibodies used were: anti-TOM20 (Santa Cruz Biotechnology, Milan, Italy), anti-LC3 (Cell Signaling, Milan, Italy). Cells were then washed with PBS (GIBCO, BE17-512F, Monza, Italy) and incubated for 1 h with labelled anti-mouse (Thermo Fisher Scientific, A11017-A21425, Monza, Italy) or anti-rabbit (Thermo Fisher Scientific, A11070-A21430-A31573, Monza, Italy) secondary antibodies. Nuclei were stained with 1 μg/mL DAPI (4’,6-diamidino-2-phenylindole) and examined under a Zeiss LSM 700 63x oil-immersion objective (CLSM700; Jena, Germany). We used ImageJ software for image analysis. We calculated the mito content as percentage of cytosolic area occupied by mitochondria with Mitophagy macro [65,66]. Co-localization measurements were made through the JACOP plugin of the NIH ImageJ software [67]. M1 manders co-localization coefficients (MCC) of mitochondria overlapping LC3. All acquisitions were performed by a blind approach in non-saturated single z-confocal. The images in Figure 1a were captured with Zoe Fluorescence Cell Imager (Biorad, Milan, Italy).

### 4.8. Statistical Analysis

All statistical analyses were performed and graphed using GraphPad Prism 6. Comparisons between two groups were analyzed using two-tailed Student’s T-test with Welch’s correction. Three or more groups comparisons were performed with one-way ANOVA with Sidak’s correction. Significance is defined as * *p* < 0.05, ** *p* < 0.01 and *** *p* < 0.001. Standard error of the mean is indicated as s.e.m.

## Supplementary Materials

The following are available online at https://www.mdpi.com/1422-0067/21/1/355/s1.

## Abbreviations

PINK1HP	TEN induced kinase 1
PRKN	Parkin RBR E3 ubiquitin ligase
SOD2	superoxide dismutase 2
TOMM20	translocase of outer mitochondrila membrane 20
VCL	vinculin
OPTN	optineurin
CALCOCO2	calcium binding and coiled-coil domain 2
BNIP3L	BCL2 interacting protein 3 like
AMBRA1	Autophagy and Beclin 1 regulator 1
FUNDC1	FUN14 domain containing 1
BCL2L13	BCL2 like 13
PHB2	Prohibitin 2
MAP1LC3A	microtubule associated protein 1 light chain 3 alpha
UTR	Un-translated Region
MDVs	Mitochondrial-Derived Vesicles
GFP	Green Fluorescent protein
Mr(K)	Relative molecular mass expressed in Kilo Dalton
O/A	Oligomycin/Antimycin A
CQ	Cloroquine
s.e.m.	Standard Error of the Mean
