# Supplementary figures and images for: miR-218 Inhibits Mitochondrial Clearance by Targeting PRKN E3 Ubiquitin Ligase
Source: Int J Mol Sci. 2020 Jan 5;21(1):355. doi: 10.3390/ijms21010355 (PMC6981953; doi:10.3390/ijms21010355)

Suppl. Figure 1

Fig.1e

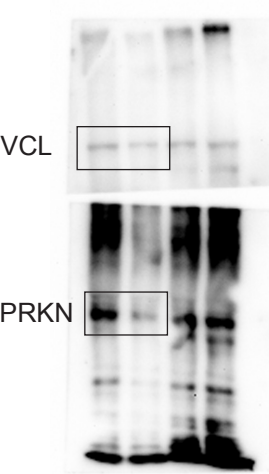

Fig.2a

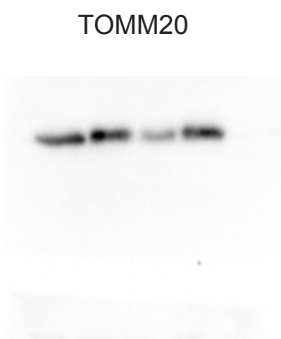

Fig.2a

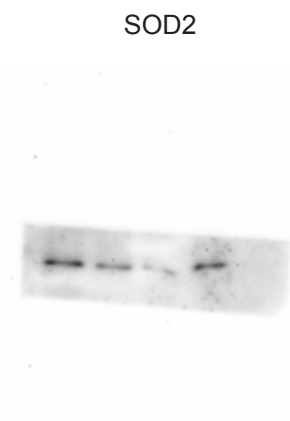

Fig.2a

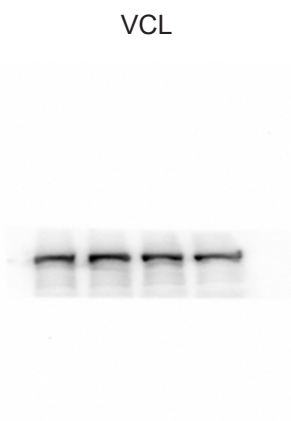

Fig.3a

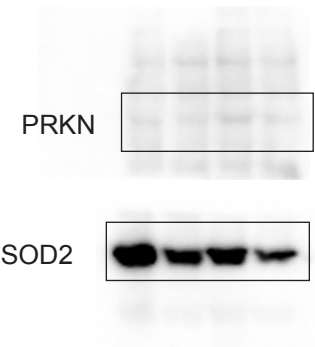

Fig.3c

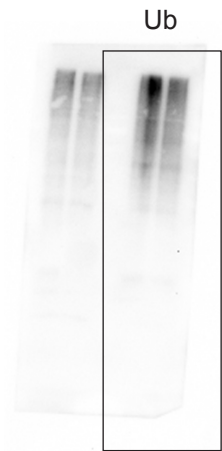

Fig.3c (same filter of tot Ub)

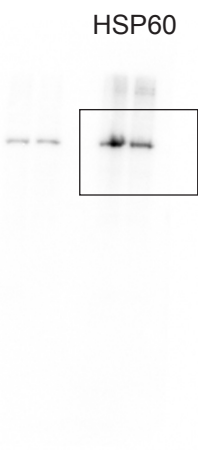

Fig.4b

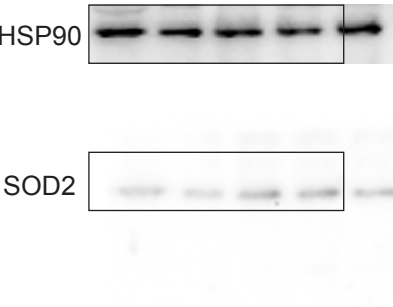

Supplement: Supplementary file 1 [file ijms-21-00355-s001.pdf]
